# Single-Cell RNA-Seq Identifies Immune Remodeling in Lungs of β-Carotene Oxygenase 2 Knockout Mice with Improved Antiviral Response

**DOI:** 10.3390/nu17213329

**Published:** 2025-10-23

**Authors:** Yashu Tang, William Lin, Xiang Chi, Huimin Chen, Dingbo Lin, Winyoo Chowanadisai, Xufang Deng, Peiran Lu

**Affiliations:** 1 Department of Nutritional Sciences, Oklahoma State University, Stillwater, OK 74078, USA; 2 Department of Physiological Sciences, Oklahoma State University, Stillwater, OK 74078, USA; 3 Center for Mitochondrial and Epigenetic Medicine and Department of Pathology and Laboratory Medicine, Children’s Hospital of Philadelphia, Philadelphia, PA 19104, USA

**Keywords:** dendritic cells, interferon signaling, lung immune heterogeneity, SARS-CoV2 viral infection, single-cell RNA sequencing (scRNA-seq), T cells

## Abstract

**Background/Objectives**: β-Carotene oxygenase-2 (BCO2) is a mitochondrial carotenoid-cleaving enzyme expressed in multiple tissues, including the lungs. While BCO2 regulates carotenoid handling, its role in shaping pulmonary immune architecture and antiviral responses is unknown. We hypothesized that BCO2 deficiency reprograms epithelial–innate circuits and alters antiviral outcomes. **Methods**: BCO2-knockout (KO) and C57BL/6J wild-type (WT) mice underwent lung single-cell RNA sequencing (scRNA-seq), immunoblotting, and intranasal SARS-CoV-2 challenge to assess cell-type heterogeneity, pathway programs (by gene set variation analysis, GSVA), and antiviral responses. **Results**: scRNA-seq resolved 14 major lung cell populations with cell-type-specific pathway shifts. Compared with WT, BCO2 KO lungs showed increased conventional dendritic cells and natural killer (NK) cells, with reductions in macrophages, B cells, and endothelial cells. In KO alveolar type II cells, GSVA indicated a stress-adapted metabolic program. Ciliated epithelium exhibited vitamin-K-responsive and axoneme-remodeling signatures with attenuated glucocorticoid and very-low-density lipoprotein remodeling. Innate lymphoid type 2 cells favored fatty acid oxidation and chromatin dynamics with reduced mitochondrial activity. NK cells were biased toward constitutive chemokine/cytokine secretion and counter-inflammatory signaling. Immunoblotting confirmed the elevated level of interferon regulatory factor-3 protein in BCO2-KO lungs. Functionally, BCO2-KO mice had improved outcomes after intranasal SARS-CoV-2 exposure. **Conclusions**: Loss of BCO2 reconfigures the pulmonary immune landscape and enhances antiviral responsiveness in mice. These findings identify BCO2 as a nutrient-linked enzyme with immunomodulatory impact and highlight cell-state changes as candidate mechanisms for improved antiviral tolerance.

## 1. Introduction

Respiratory health requires a finely tuned equilibrium of tolerance to persistent aeroantigens and rapid antiviral readiness against pathogens [1]. This balance, an aspect of immune priming and resilience, is set by epithelial metabolic tone and coordinated epithelial–immune communication [2]. Defining molecular levers that tune these basal set-points is essential for understanding how the lung resists infection while limiting tissue damage, and it provides a nutrition-relevant opportunity to modulate barrier function.

β-Carotene oxygenase-2 (BCO2) is a mitochondrial carotenoid-cleaving enzyme classically studied in nutrient metabolism, acting on provitamin A carotenoids and xanthophylls [3,4]. Beyond carotenoid processing, BCO2 localizes to the inner mitochondrial membrane and has been implicated in regulating respiration, limiting reactive oxygen species (ROS), and maintaining redox balance [3,5,6]. Additional work links BCO2 to inflammatory signaling and metabolic adaptation [5,6]. Notably, genome-wide association studies identify the IL18–BCO2 loci as a regulatory hotspot associated with circulating IL-18 and immune pathways [7,8]. Together, these observations position BCO2 as a candidate metabolic–immune regulator linking dietary carotenoid biology, mitochondrial homeostasis, and inflammation. Most evidence derives from the intestine, liver, and adipose tissue; its role in the lung remains poorly defined.

The lung is a tolerogenic, yet vigilant mucosal organ composed of diverse epithelial, immune, endothelial, and stromal populations that coordinate gas exchange with immune defense and repair [9]. Because inhaled antigens and commensal signals are continuous, pulmonary homeostasis requires tolerance to avoid excessive inflammation while preserving rapid antiviral responses [10]. Disruption of cellular identity, metabolic poise, or intercellular communication contributes to acute and chronic lung pathology [11]. During infection, epithelial and immune cells undergo profound transcriptional and metabolic remodeling; when dysregulated, these responses can amplify tissue injury and impair repair [12,13]. Understanding how nutrient-linked mitochondrial processes calibrate this tolerance–vigilance balance is an unmet need.

At the respiratory barrier, epithelial and resident immune cells integrate multiple pattern-recognition pathways to balance tolerance with rapid antiviral readiness [14]. Cytosolic DNA is detected by cyclic GMP–AMP synthase (cGAS), which produces cyclic GMP–AMP (cGAMP) to activate the stimulator of interferon genes (STING) at the endoplasmic reticulum (ER)–Golgi interface; STING recruits TANK-binding kinase 1 (TBK1) to phosphorylate interferon regulatory factor 3 (IRF3), inducing type I/III interferons and interferon-stimulated genes (ISGs) while also engaging Nuclear Factor kappa-light-chain-enhancer of activated B cells (NF-κB) [15,16]. In parallel, viral RNA is sensed by retinoic acid-inducible gene I (RIG-I) and melanoma differentiation-associated protein 5 (MDA5), which signal via the mitochondrial antiviral-signaling protein (MAVS) on the outer mitochondrial membrane to activate TBK1–IRF3 and NF-κB [17,18]. Within this framework, IRF3 serves as a central transcriptional hub that coordinates antiviral gene programs and sets basal preparedness. Because MAVS is mitochondrial and IRF3/STING outputs are redox-sensitive, innate signaling is tightly coupled to organelle metabolism [19]. Thus, enzymes, such as BCO2 which influence mitochondrial respiration and reactive oxygen species (ROS)/redox tone, provide a plausible lever to tune the cGAS–STING and RIG-I/MDA5–MAVS pathway set-points in the lung epithelium, potentially aligning epithelial tolerance with rapid antiviral signaling without tipping into excessive inflammation.

To resolve these pathway-level interactions at cellular resolution, bulk transcriptomics is insufficient because it obscures cell-type-specific programs and rare states that are critical in barrier tissues. Single-cell RNA sequencing (scRNA-seq) provides unbiased resolution of cell types and their transitions [20]. By detecting dynamics that bulk profiling cannot, it is particularly useful in the lung, a tissue in which epithelial–immune crosstalk regulates tolerance, injury responses, and antiviral preparedness [21,22]. Complementing scRNA-seq, gene set variation analysis (GSVA) aggregates transcript-level changes into pathway activities, providing cell-type-resolved views of metabolic and innate-immune rewiring [23].

Here, we used scRNA-seq to compare whole-lung cellular landscapes in BCO2 knockout (KO) and C57BL/6J wild-type (WT) mice and applied GSVA to resolve pathway-level changes by cell type [11]. We further validated innate signaling biochemically (by determining IRF3) and assessed challenge-responsive antiviral readouts in a SARS-CoV-2 exposure model. Our mechanistic goal is to define how BCO2 tunes epithelial mitochondrial and innate-signaling set points consistent with tolerance and antiviral readiness; we do not assess histopathology, instead focusing on a single-cell/pathway framework for epithelial–immune orchestration.

## 2. Materials and Methods

### 2.1. Animals and Diets

C57BL/6J wild-type (WT or B6) mice and age- and sex-matched littermates of BCO2 knockout (KO) mice (C57BL/6J isogenic background) were obtained from our in-house colonies generated by backcrossing BCO2^+/−^ breeders on a standard AIN-93M diet with ad libitum access to food and water [24]. Animals were housed in the Oklahoma State University (OSU) AAALAC-accredited animal facility under controlled conditions: temperature maintained at 22 ± 2 °C, 40–60% relative humidity, and a 12 h light/12 h dark cycle. At 8 weeks of age, mice were euthanized for lung cell isolation and immunoblotting. SARS-CoV-2 challenge was conducted in aged mice as described below. Pain and distress were minimized using approved anesthetics and analgesics in accordance with NIH and institutional animal care guidelines and the Institutional Animal Care and Use Committee (IACUC) protocol. All animal experiments and procedures were approved by the Oklahoma State University IACUC (ACUP #HS-20-75 and #22-55).

### 2.2. Single Cell Isolation and Fixation

All mice were euthanized using a ketamine/xylazine cocktail after 3 h fasting. Whole-lung tissues were harvested and rinsed in PBS supplemented with 2% fetal bovine serum (FBS), and single cells were isolated using the protocol as described (https://www.stemcell.com/mouse-lung-dissociation-into-single-cell-suspension.html (accessed on 17 June 2024)). Briefly, tissue samples were minced and digested at 37 °C for 20 min in RPMI supplemented with collagenase/Hyaluronidase (Stemcell #07912, Stemcell Technologies, Cambridge, MA, USA) and 0.15 mg/mL DNase I (STEMCELL #07900). The suspension was filtered through 70 µm cell strainers, then treated with ammonium chloride solution for 10 min at room temperature to remove red blood cells. The cells were washed in PBS + 2% FBS and used for downstream assays. Trypan blue was used to test cell viability. Samples with cell viability greater than 80% were fixed using the EvercodeTM cell fixation v2 (ECF2101, Parse Biosciences, Seattle, WA, USA) and then stored at −80 °C.

### 2.3. Probe Hybridization and Library Construction

The EvercodeTM Mini v2 kit (ECW02110, Parse Biosciences) was used for probe hybridization and library construction, exactly following the protocols as provided. Five thousand cells per fixed lung sample were subjected to sequencing at 50,000 reads per cell, which was completed in the Cancer Center at University of Colorado Anschutz Medical Campus (Aurora, CO, USA).

### 2.4. Processing and Quality Control of scRNA-Seq Data

The same processing and analysis pipeline was used across both samples as we previously reported [8]. Read processing was performed using the Cellranger pipeline (version 7.0.0), which performed read alignment, barcode and unique molecular identifier (UMI) processing, and data aggregation and normalization. The reads were aligned to 10X Reference Genome GRCm38. Then, the Cellranger output was read in and converted into a Seurat object using the Read10X and CreateSeuratObject functions in R. The parameters used for cell quality control were min_cells = 3, nFeature_RNA  >  200, percent.mt < 25.

After removing low-quality cells, the gene expression levels for each cell were normalized with the NormalizeData function in Seurat (v5). ScaleData function was used to linearly regress out variations sourced from different numbers of unique molecular identifiers (UMIs) and mitochondrial gene expression. Highly variable genes were subsequently identified by the FindVariableFeatures function. The scaled data was then subjected to PCA running. Then, the 15 most significant principal components were selected via principal component analysis (PCA) and were then used to perform UMAP dimensionality reduction. Cell clustering was performed using the ‘FindNeighbors’ and ‘FindClusters’ functions in Seurat. Classical marker genes were used to annotate the cell type of each cluster.

### 2.5. Gene Set Variation Analysis (GSVA) Analysis

To assess functional differences between samples, gene set variation analysis (GSVA) was applied using the GSVA R package (version 1.52.3) with default settings [23]. Gene sets were retrieved from the Molecular Signatures Database (MSigDB). Differential pathway activity between KO and WT within each cell type was assessed by linear models with sample as the unit, controlling for sex where applicable; *p* values were FDR-adjusted (Benjamini–Hochberg).

### 2.6. Key Visualization and Annotation

Seurat (VlnPlot, DotPlot, DimPlot) was used to generate violin, dot, and UMAP plots; gplots (balloonplot) was used for balloon plots; and ggplot2 (ggplot with geom_bar) was used for bar plots.

### 2.7. Immunoblotting

Whole-lung tissues (~100 mg/mouse) from WT and KO mice were homogenized in 1 mL cell lysis buffer containing 20 mM Tris (pH 7.5), 0.5 mM EDTA, 0.5 mM EGTA, 0.5% Triton X-100, and 1% protease/phosphatase inhibitor cocktail, as previously described [5,6]. Proteins were separated on 8% SDS–PAGE gels and transferred to PVDF membranes (Bio-Rad, catalog #1620177, Hercules, CA, USA) for immunoblotting. Pierce ECL Western blotting Substrate (catalog no. 32109; Thermo Fisher Scientific, Waltham, MA, USA) was used for chemiluminescent detection. Band intensities were quantified using ImageJ (NIH, Bethesda, MD, USA). Primary and secondary antibodies were obtained from Cell Signaling Technology (Cambridge, MA, USA) (IRF3 (D83B9), catalog #4302 at 1:1000; β-actin, catalog #4967 at 1:2000; and anti-rabbit IgG, HRP-linked antibody, catalog #7075 at 1:1000).

### 2.8. Mouse Infection of SARS-CoV-2

Twenty-six- to twenty-eight-week-old BCO2 KO mice and their age- and sex-matched WT littermates were briefly anesthetized with isoflurane and intranasally (i.n.) inoculated with 10,000 plaque-forming units (PFU) of the mouse-adapted SARS-CoV-2-N501YMA30 (a gift from Dr. Stanley Perlman, University of Iowa) in a total volume of 50 μL Dulbecco’s Modified Eagle Medium (DMEM) [25]. Mouse body weight and clinical condition were monitored daily. Animals that reached a humane endpoint of ≥25% body weight loss were humanely euthanized and considered as deaths for survival analysis. Using GraphPad Prism 10.5.0, body weight loss curves were generated by normalizing each mouse’s daily body weight to its weight on the day of infection, and the mean ± SEM for each group was plotted over time. Survival curves were constructed using the Kaplan–Meier method, with the day of infection designated as day 0.

### 2.9. Statistical Analysis

Group differences were assessed with two-tailed Student’s *t*-tests (unpaired unless noted; Welch’s correction for unequal variances), with statistical significance set at *p* < 0.05. Sample sizes were various; *n* = 4–8.

## 3. Results

### 3.1. Single-Cell RNA-Seq Data Quality Control, Clustering, and Annotation

**Figure 1:**
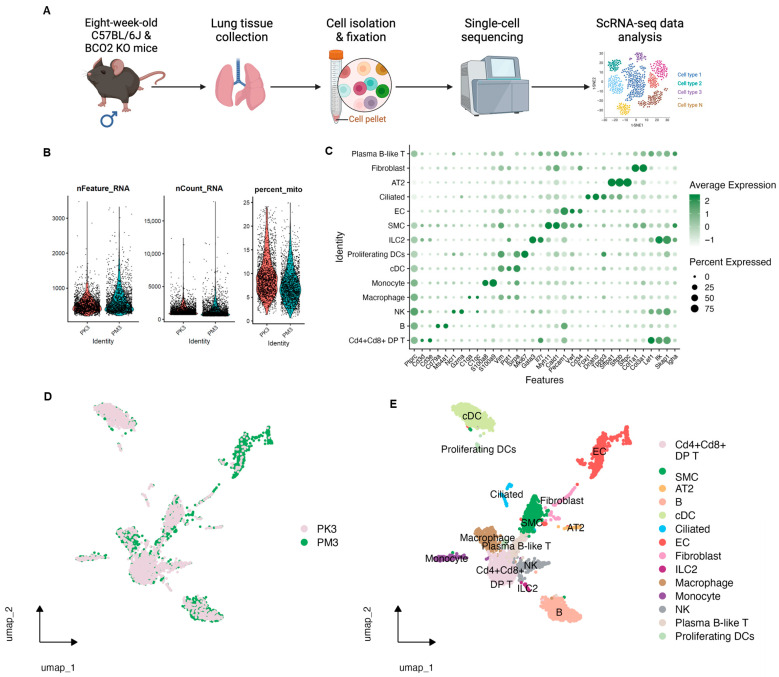
Single cell atlas of mouse lung tissues (**A**) scRNA-seq workflow. Wild-type (WT) and BCO2 KO (KO) mice were sacrificed at 8 weeks of age, and their lung tissues were harvested for single cell isolation. *n* = 1 (pooled)/group. (**B**) Quality control metrics of nFeature_RNA (**left**): number of unique genes detected per cell; nCount_RNA (**middle**): total RNA molecule counts per cell and percent_mito (**right**): proportion of mitochondrial gene expression. (**C**) Dotplot showing expression levels for canonical markers of all 14 detected cell populations. The intensity of expression is indicated by green coloring. (**D**,**E**) Uniform manifold approximation and projection (umap) representation of distribution of cells by sample group (**D**) and all 14 distinct detected cell types (**E**). PK3: BCO2 KO group; PM3: wild-type group; Cd4 + Cd8 + DP T: Cd4 + Cd8 + double-positive T cells; SMCs: smooth muscle cells; cDCs: conventional dendritic cells; Ciliated: ciliated (epithelial) cells; ECs: endothelial cells; ILC2: type 2 innate lymphoid cells; NK: natural killer cells; Proliferating DCs: proliferating dendritic cells. The workflow chart was prepared using BioRender software (https://www.biorender.com/, accessed on 15 August 2024).

In this study, ~5000 single cells from each condition (male WT and BCO2 KO) were isolated from mouse lungs, pooled, and then subjected to library construction and sequencing. Raw FASTQ files were processed to gene–cell count matrices and underwent standard quality control (Figure 1A). As shown in Figure 1B, the distributions of detected genes per cell (nFeature_RNA), total UMIs per cell (nCount_RNA), and mitochondrial transcript percentage (percent.mt; ~2–25%) indicated high-quality data suitable for downstream analyses. After QC, 1832 WT (PM3) and 1916 KO (PK3) cells were retained.

**Figure 2:**
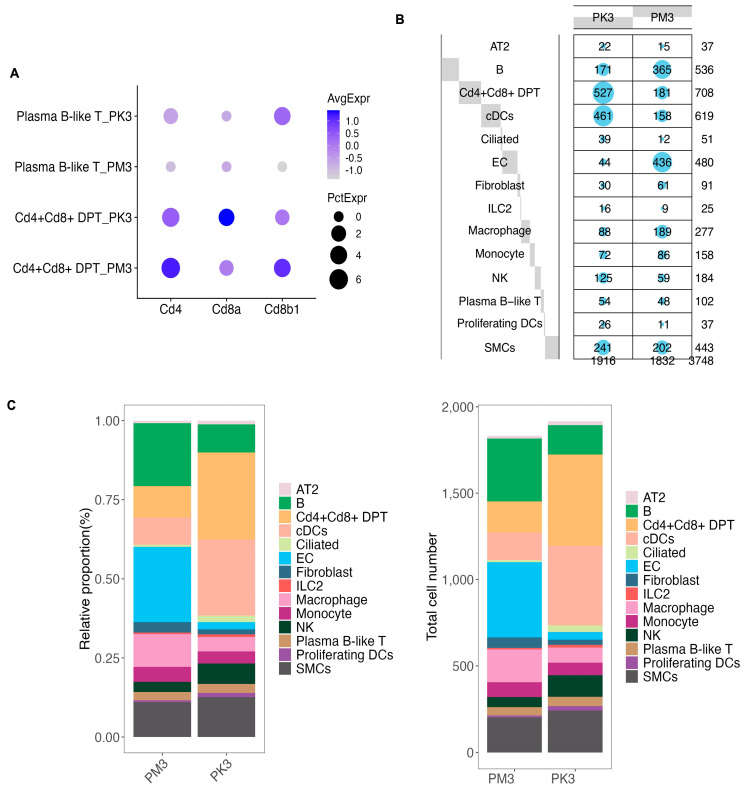
Comparative analysis of cell composition in BCO2 KO and wild-type (WT) groups. (**A**) DotPlot showing the expression of T cell marker genes of Cd4, Cd8a, and Cd8b1 across the detected T cell lineages in each group. Dot size represents the percentage of cells expressing the gene, while color intensity indicates average expression levels. Cd4 + Cd8+ DP T_PK3: Cd4 + Cd8+ double-positive T cells from the BCO2 KO group; Cd4 + Cd8+ DP T_PM3: Cd4 + Cd8+ double-positive T cells from the wild-type group; Plasma B-like T_PK3: Plasma B-like T cells from the BCO2 KO group; Plasma B-like T_PM3: Plasma B-like T cells from the wild-type group. (**B**) Balloon plot illustrating the frequency of each cell type in the wild-type (PM3) and BCO2 KO (PK3) groups.Marker size (gray squares/blue dots) indicates the percentage of each cell type. (**C**) Bar chart of the relative proportions (%) of different cell types (**left panels**) and total number of cells per cell type (**right panels**) in wild-type (PM3) and BCO2 KO (PK3) mice. Cd4 + Cd8 + DP T: Cd4 + Cd8 + double-positive T cells; SMC: smooth muscle cells; cDC: conventional dendritic cells; Ciliated: ciliated epithelial cells; EC: endothelial cells; ILC2: type 2 innate lymphoid cells; NK: natural killer cells; Proliferating DCs: proliferating dendritic cells.

Unsupervised clustering and UMAP embedding identified 14 major cell populations using canonical lung markers (Figure 1C–E). Cell-type identities were consistent across genotypes and showed substantial overlap between WT and KO embeddings. The atlas included structural cells, such as alveolar type II (AT2), ciliated epithelium, fibroblasts, smooth muscle cells (SMCs), and endothelial cells (ECs), and immune cells, including macrophages, monocytes, natural killer (NK) cells, innate lymphoid cells type 2 (ILC2), B cells, plasma B-like cells, CD4/CD8 double-positive (DP) T cells, conventional dendritic cells (cDCs), and proliferating DCs (Figure 2A).

Cell number summaries and normalized frequencies (balloon plot) are shown in Figure 2B,C. When subcluster counts were normalized to total cells per sample, KO lungs (PK3) exhibited higher proportions of CD4/CD8 DP T cells, cDCs, ciliated cells, ILC2, NK cells, and proliferating DCs, with lower proportions of B cells, ECs, and macrophages compared to WT (PM3). This compositional shift established the framework for the cell-type-resolved pathway analyses described below.

### 3.2. GSVA Reveals Cell-Type-Specific Pathway Shifts in BCO2 KO

**Figure 3:**
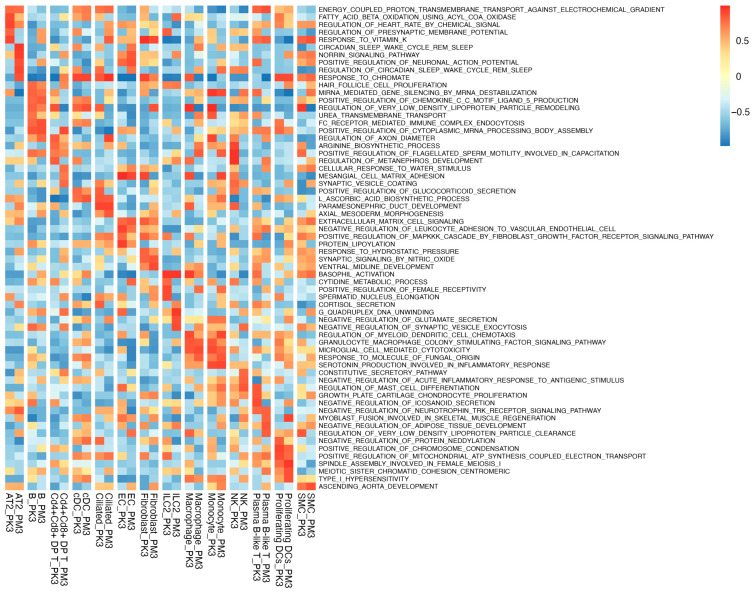
Heatmap of gene set variation analysis (GSVA) of Gene Ontology biological process gene sets. Each row represents a distinct gene set, and each column represents a specific cell type from an experimental group (PM3: wild-type; PK3: BCO2 KO). The GSVA score, represented by the color scale, indicates the level of variation in gene set expression. Cd4 + Cd8+ DP T: Cd4 + Cd8+ double-positive T cells; SMC: smooth muscle cell; cDC: conventional dendritic cell; Ciliated: ciliated epithelial cell; EC: endothelial cell; ILC2: type 2 innate lymphoid cells; NK: natural killer cells; Proliferating DCs: proliferating dendritic cells.

GSVA revealed coordinated epithelial stress–metabolic adaptation and innate pathway remodeling in BCO2 KO (Figure 3). Specifically, for AT2 cells, circadian/oxidative-stress and Norrin/Wnt programs were enriched, while peroxisomal fatty acid oxidation and mitochondrial protein lipoylation were attenuated; proton-coupled oxidative phosphorylation was largely stable, consistent with metabolic substrate rerouting rather than loss of respiratory capacity. For ciliated cells, vitamin-K-responsive and axoneme/cytoskeletal-remodeling signatures were enriched, with attenuated glucocorticoid signaling, very-low-density lipoprotein cholesterol (VLDL) remodeling, and negative regulation of neddylation, indicative of structural adaptation and endocrine de-sensitization. For immune cell types, the results revealed that fatty acid oxidation and chromatin/DNA-remodeling programs were enriched in ILC2 cells, with reduced mitochondrial ATP-coupled electron transport and neuroexcitable modules, supporting a lipid-powered, reparative state. Furthermore, despite higher abundance of NK cell populations, the pathway profiles favored constitutive chemokine/cytokine secretion and counter-inflammatory signaling, with diminished arginine/nitric oxide (NO) and degranulation modules, emphasizing milieu modulation over direct cytotoxicity. The numbers of cDCs increased (including a proliferating subset), while baseline GSVA remained largely unchanged, consistent with the expansion of steady-state cDCs; macrophages, B cells, and ECs decreased in KO lungs.

### 3.3. BCO2 Deficiency Primes IRF3 Signaling and Improves SARS-CoV-2 Outcomes

**Figure 4:**
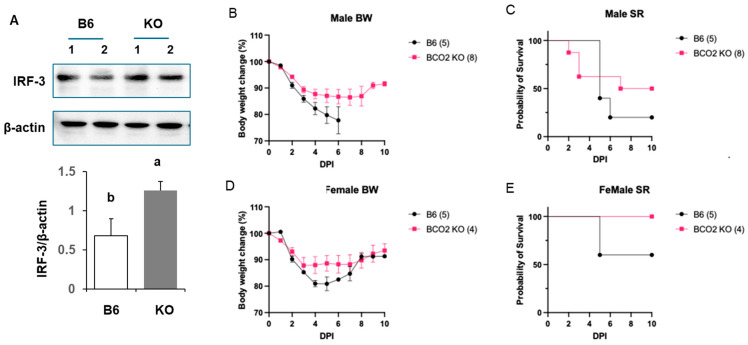
Enhanced IRF3 protein levels and improved SARS-CoV-2 outcomes in KO mice. (**A**) Elevated protein levels of interferon regulatory factor 3 (IRF-3) in the lung of male BCO2 knockout (KO) mice, compared to the littermate wild-type C57BL/6J (B6) mice. Quantified data is shown. N = 4; representative images shown. Lowercase letters denote groups that differ significantly (*p* < 0.05). (**B**–**E**) Body weight loss and survival rate (SR) in BCO2 knockout (KO) mice and the littermate wild-type C57BL/6J (B6) mice exposed to SARS-CoV-2 intranasal infection. (**B**) Body weight loss in male mice. (**C**) Survival rate in males. (**D**) Body weight loss in females. (**E**) Survival rate in females. Animal numbers are indicated in parentheses (*n* = 4–8). BW, body weight; DPI, days post-infection; SR, survival rate.

Immunoblotting of whole-lung lysates showed increased IRF3 protein levels in male BCO2 KO, compared to age- and sex-matched WT littermates (Figure 4A). The result suggested baseline activation of the IRF3 innate antiviral axis in BCO2-deficient lungs [26,27].

To assess functional consequences, mice were intranasally infected with mouse-adapted SARS-CoV-2, and body weight and survival rate were monitored (Figure 4B–E). In males, KO animals had attenuated weight loss, most evident at 3–6 days post-infection (DPI), and began recovering by DPI 8–9, whereas WT continued to decline (Figure 4B). The survival rate was higher in KO males (~50% at DPI 9) versus WT (~20%) (Figure 4C). In females, early weight loss (DPI 1–DPI 3) was similar between genotypes, but KO mice recovered faster, reaching ~95% of baseline by DPI 9 (Figure 4D); the survival rate was 100% in KO females, whereas the WT survival rate was ~60% by DPI 6 (Figure 4E). Together, enhanced IRF3 activation at baseline and improved survival rates supported a model in which BCO2 deficiency establishes an infection-ready lung state.

## 4. Discussion

This study defines a baseline lung configuration associated with BCO2 loss that integrates epithelial metabolic rewiring with coordinated innate remodeling. The single cell atlas shows a shift toward orchestration and priming, a relative expansion of NK cells and cDCs, and reduced macrophages, B cells, and endothelial cells, accompanied by cell-state programs consistent with stress-adapted AT2 metabolism, ciliated structural remodeling, and restrained NK effector modules [28,29]. Together with elevated IRF3 protein levels, these features align with a pre-alert interferon axis and a tissue milieu biased toward coordinate-and-protect rather than kill-centric responses to SARS-CoV-2 infection [30,31,32,33].

At the compositional level, the BCO2 KO redistribution supports a redox-tuned epithelial–innate crosstalk model. Given BCO2’s localization at the inner mitochondrial membrane and roles in respiration and ROS/redox buffering, variation in BCO2 activity would be expected to influence pattern-recognition signaling thresholds and cytokine set-points [34]. The relative increase in cDCs (including a proliferative subset) suggests enhanced capacity for antigen handling/instruction, whereas the decrease in macrophages and B cells indicates less emphasis on lipid/particle clearance and humoral niche maintenance at baseline [30,31]. We consider this as pre-positioning for efficient orchestration while minimizing unnecessary tissue-damaging effector deployment.

Pathway analysis reinforced this coordinated bias. GSVA (of male lungs) identified cell type-specific programs. AT2 cells exhibited substrate rerouting and stress-adapted metabolism; ciliated epithelium showed axonemal/structural remodeling with dampened endocrine lipid cues; ILC2 adopted lipid-powered, chromatin-dynamic states; NK cells, despite numerical expansion, favored secretory/counter-inflammatory profiles over classic cytotoxicity; and cDCs expanded in a poised steady state [23,35,36,37]. Elevated IRF3 suggests interferon priming at baseline, helping drive these programs and temper inflammatory magnitude [32,33]. Mechanistically, such tuning is compatible with BCO2-dependent mitochondrial/redox control of RIG-I/MDA5–MAVS and cGAS–STING axes converging on TBK1–IRF3.

Following SARS-CoV-2 exposure, Bco2 KO mice (both sexes) lost less weight and had higher survival, with a stronger effect in females. Although we did not quantify lung histopathology, the single-cell and signaling data point to three non-exclusive mechanisms of improved tolerance: (1) front-line epithelial resilience, AT2 substrate rerouting, and ciliated remodeling that may support barrier and mucociliary function [38,39]; (2) orchestrated innate readiness and expanded NK/cDC compartments with restrained effector amplitude, limiting bystander injury [40,41]; and (3) baseline IRF3 priming that may enable earlier antiviral engagement with less tissue damage [42]. Future work should test whether these programs are associated with reduced epithelial denudation, edema, or diffuse alveolar damage, and whether they preserve cilium integrity and surfactant homeostasis during infection.

This study was designed to resolve baseline and early-response mechanisms. Discovery assays, single-cell profiling (scRNA-seq/GSVA), and IRF3 immunoblotting were performed in males to control hormonal variability, whereas the SARS-CoV-2 challenge cohort included both sexes. Accordingly, our cellular and pathway inferences are male-derived, while survival and weight-loss outcomes are sex-stratified (Figure 4). The stronger benefit observed in females is hypothesis-generating and consistent with sex-dependent calibration of antiviral set points via BCO2-mediated mitochondrial/redox control. Key limitations include the absence of viral burden, histopathology, cytokine protein kinetics, spatial niche readouts (airway vs. parenchyma), the use of global rather than cell-specific BCO2 loss, and the fact that GSVA can be influenced by gene-set composition and scoring choices [43]. To convert mechanistic alignment into causal evidence, future work will focus on (i) sex-aware molecular profiling (including female datasets), (ii) spatial and temporal resolution of lung responses during infection, and (iii) causal tests using cell-type-specific genetics and targeted perturbations to link BCO2-dependent mitochondrial control to tissue injury, repair, and outcome.

## 5. Conclusions

Our study repositions BCO2 from a carotenoid-cleaving enzyme to a mitochondrial regulator of lung innate set points, establishing a mechanistic link between nutrition, organelle redox control, and mucosal immunity. At single-cell resolution, BCO2 loss shifts the lung toward an orchestration/priming configuration (NK/cDC bias with coordinated epithelial programs) and aligns with elevated IRF3 consistent with basal interferon readiness; in vivo, this state improves challenge outcomes, with a stronger benefit in females. Collectively, these findings define a testable model in which BCO2-dependent mitochondrial/redox tuning balance tolerance and vigilance at the respiratory barrier and identify BCO2 as an actionable node for precision nutrition and immunometabolic interventions. While detailed pathology and viral load measures were beyond scope, the convergence of composition, state, signaling, and survival advances the field by (i) revealing a nutrient-linked control layer over epithelial–innate crosstalk, (ii) providing a single-cell framework to parse sex-dependent resilience, and (iii) setting clear, causal hypotheses for cell-type-specific and diet-informed strategies to enhance antiviral readiness with minimal immunopathology.

## Data Availability

The original contributions presented in this study are included in the article. Further inquiries can be directed to the corresponding authors.

## Abbreviations

The following abbreviations are used in this manuscript:

AT2	Alveolar type II cell
BCO2	β-carotene oxygenase-2
cDC	Conventional dendritic cell
cDNA	Complementary DNA
cGAS	cyclic GMP–AMP synthase
cGAMP	cyclic GMP–AMP
DP	Double-positive
EC	Endothelial cell
ER	Endoplasmic reticulum
ETC	Electron transport chain
GSVA	Gene set variation analysis
ILC2	Innate lymphoid cell type 2
IRF3	Interferon regulatory factor 3
ISG	Interferon-stimulated gene
KO	Knockout
MAVS	Melanoma differentiation-associated protein 5
MDA5	Mitochondrial antiviral-signaling protein
NF-κB	Nuclear Factor kappa-light-chain-enhancer of activated B cells
NK	Natural killer cell
OXPHOS	Oxidative phosphorylation
RIG-I	Retinoic acid-inducible gene I
SARS-CoV-2	Severe acute respiratory syndrome coronavirus 2
SMC	Smooth muscle cell
STINIG	Stimulator of interferon genes
TBK1	TANK-binding kinase 1
UMAP	Uniform Manifold Approximation and Projection
UMI	Unique molecular identifier
VLDL	Very-low-density lipoprotein
WT	Wild-type
